# Patients Lacking a KIR-Ligand of HLA Group C1 or C2 Have a Better Outcome after Umbilical Cord Blood Transplantation

**DOI:** 10.3389/fimmu.2017.00810

**Published:** 2017-07-13

**Authors:** Carmen Martínez-Losada, Carmen Martín, Rafael Gonzalez, Bárbara Manzanares, Estefania García-Torres, Concha Herrera

**Affiliations:** ^1^Department of Hematology, Reina Sofía University Hospital/Instituto Maimónides de Investigación Biomédica (IMIBIC), University of Córdoba, Córdoba, Spain; ^2^Department of Inmunology, Reina Sofía University Hospital, Córdoba, Spain

**Keywords:** KIR, KIR-ligand, UCB-transplantion, leukemia, relapse

## Abstract

Donor natural killer (NK) cells can destroy residual leukemic cells after allogeneic hematopoietic stem cell transplantation. This effect is based on the interaction of killer-cell immunoglobulin-like receptors (KIR) of donor NK cells with ligands of the major histocompatibility complex found on the surface of the target cells. HLA-C1 subtypes provide the ligand for KIR2DL2 and KIR2DL3 and the HLA-C2 subtypes for KIR2DL1. We have studied the probability of relapse (PR) after single-unit unrelated cord blood transplantation (UCBT) in relation to the potential graft-vs.-leukemia effect mediated by NK cells present in the umbilical cord blood (UCB) by analyzing KIR-ligand and HLA-C typing of the receptor. Data from 33 consecutive patients given a single unit UCBT were included. We have considered two groups of patients based on the absence or the presence of one of the C-ligands for inhibitory KIR and the incompatibility HLA-C1/2 between UCB and patients. Group 1 (*n* = 21): the patient lacks a C-ligand for inhibitory KIR present in UCB NK cells, i.e., patients homozygous C1/C1 or C2/C2. Group 2 (*n* = 12): patients heterozygous C1/C2 in which KIR-mediated graft-vs.-leukemia effect is not expected (presence of both C ligands for inhibitory KIR in the receptor). With a median follow-up post-UCBT of 93 months, patients with absence of a C-ligand for inhibitory KIRs (Group 1) showed a lower actuarial PR than patients with both C-ligands (group 2): 21 ± 10 vs. 68 ± 18% at 2 year and 36 ± 13 vs. 84 ± 14% at 5 years (*p* = 0.025), respectively. In patients with acute lymphoblastic leukemia, the 2-year PR was 36 ± 21% for group 1 and 66 ± 26% for 2 (*p* = 0.038). Furthermore, group 1 had a lower incidence of grades II–IV acute graft-vs.-host disease (*p* = 0.04). In the setting of UCBT, the absence of a C-ligand (C1 or C2) of inhibitory KIR in the patient is associated with lower PR, which is probably due to the graft-vs.-host leukemia effect caused by UCB NK cells that lack a ligand for the inhibitory KIR 2DL1/2DL2/2DL3.

## Introduction

Allogeneic hematopoietic stem cell transplantation (HSCT) is a well-established therapy for a considerable number of patients with acute leukemia. Besides cytotoxic therapy, graft-vs.-leukemia (GvL) effect is one of the most important factor for the success of HSCT. Apart from T cells, alloreactive natural killer (NK) cells have shown that they can induce GvL following HSCT (1).

Natural killer cells are lymphocytes that have an important role in innate immune responses, particularly in regulating the defense to viral infections and cancer development. They serve important functions in immune surveillance against hematologic malignancies (2, 3). Therefore, NK cells have receptors on their surface, which are able to identify ligands of the major histocompatibility complex (MHC) found on the surface of the target cells. These receptors may inhibit or activate the effector function of NK cells (4).

In humans, a relatively large family of NK cell receptors for MHC is represented by killer-cell immunoglobulin-like receptors (KIRs). These are characterized by two (KIR2D) or three (KIR3D) extracellular immunoglobulin domains. In addition, they have either short (S) or long (L) intracytoplasmic tails, which transduce activating or inhibitory signals, respectively. Different KIR genes have been identified; KIR2DL1-3, KIR2DL5, KIR3DL1-3 are inhibitory; KIR2DS1-5 and KIR3DS1 are activating, KIR2DP1 and KIR3DP1 are pseudo genes, and KIR2DL4 has both inhibitory and activating properties (4). KIRs recognize allotypic determinants (“KIR ligands”) shared by certain HLA class I allele groups. KIR2DL1 recognizes HLA-C2 alleles with Lys^80^ residue; KIR2DL2 and KIR2DL3 recognize HLA-C1 with an Asn^80^ residue; KIR3DL1 is the receptor for HLA-B alleles sharing the Bw4 supertypic specificity. Finally, KIR3DL2 was shown to function as a receptor for HLA-A3/-A11 alleles when bound to Epstein–Barr virus (EBV) peptides (5).

The role of KIR and KIR ligands in the context of HSCT has been investigated (6). Some studies have found that KIR ligand compatibility or incompatibility influence the prognosis of HSCT between unrelated individuals (7). The absence of an HLA-ligand in the patient and the presence of the corresponding inhibitory KIR in the donor generate a potential NK alloreactivity against the patient target cells, resulting in alloreactivity in the graft-vs.-host (GVH) direction. Conversely, the presence of an epitope of HLA ligand for KIR in the patient and its absence in the donor will result in alloreactivity in the rejection direction (8, 9).

Umbilical cord blood (UCB) units from unrelated donors constitute a well-established source of stem cells for HSCT in patients with leukemia. The main advantages are the rapid availability and the possibility of performing HLA-mismatched transplantations due to a relatively low risk of severe graft-vs.-host disease (GVHD) that increases the probability of finding a suitable donor (10).

Because if the extensive genetic variability of KIR and/or their HLA ligands and the importance of their combinations in the response of NK cells, we have studied the possible effect of GvL mediated by NK cells present in UCB according to the HLA-C typing of the patients. We explored how a model based only on recipient HLA typing could predict donor NK alloreactivity through KIR ligand status, because 96% of them have inhibitory KIRs for HLA-C1 and C2 (11). For this purpose, UCB NK alloreactivity was analyzed in relation to the presence or absence of HLA-C1 and C2 ligands in the receptor and the consequent lack of binding of their corresponding inhibitory KIR.

## Materials and Methods

### Patients

A total of 33 patients (16 males, 17 females) were included in this retrospective study. All of them underwent an HSCT from a single UCB unit at the Hematopoietic Transplantation Unit of Reina Sofía University Hospital, Córdoba, Spain, between September 2002 and October 2011. To enter the study, it was necessary that each patient had a clinical follow-up of at least 6 months and have, both the patient and the UCB unit, allele-level molecular typing for HLA-C, together with HLA typing for HLA-A, HLA-B, and DRB1. Median age is 11 years, range 1–48 years. Eighteen patients were diagnosed with acute lymphoblastic leukemia (ALL), 13 with acute myeloid leukemia (AML), and 2 with other diagnostics. The characteristics of the patients are showed in Table 1.

**Table 1:** Patient and transplantation characteristics.

	Entire population	Group 1	Group 2	
Number of patients	33	21	12	
Median age (years)	11 (1–48)	10 (3–40)	15 (1–48)	*p* = 0.5
Median follow-up	93 months	95 months	71 months	*p* = 0.1
Sex (male/female)	16/17	8/13	8/4	*p* = 0.1
Diagnosis; *n* (%)				*p* = 0.4
AML	14 (39%)	10 (48%)	3 (25%)	
ALL	18 (55%)	10 (48%)	8 (67%)	
Others	2 (6%)	1 (4%)	1 (8%)	
Status at transplant				*p* = 0.5
1st CR	14 (43%)	6 (29%)	8 (67%)	
2nd CR	14 (43%)	12 (57%)	2 (16%)	
Refractory disease	5 (14%)	3 (14%)	2 (16%)	
High molecular/cytogenetic risk^a^	12 (36%)	9 (42.8%)	3 (25%)	*p* = 0.5
TNC-infused (median ×10^7^/kg)	3 (0.62–7.30)	3 (0.8–7.30)	2.6 (0.62–5.88)	*p* = 0.5
CD34+ infused (median ×10^5^/kg)	2.4 (0.02–9.40)	2.1 (0.02–9.4)	2.09 (0.19–8)	*p* = 0.6
Number of HLA disparities^b^				*p* = 0.3
6/6 match	2 (6%)	2 (9%)	0	
5/6 match	14 (42%)	12 (57%)	7 (58%)	
4/6 match	17 (52%)	7 (34%)	5 (42%)	
Conditioning regimen; *n* (%)				*p* = 0.5
Myeloablative conditioning	29 (88%)	18 (86%)	11 (92%)	
Reduced-intensity conditioning	4 (12%)	3 (14%)	1 (8%)	
ATG	33 (100%)	21 (100%)	12 (100%)	
GVHD prophylaxis				*p* = 0.2
CSA + prednisone	25 (76%)	14 (67%)	11 (92%)	
CSA + MMF	8 (24%)	7 (33%)	1 (8%)	

*AML, acute myeloblastic leukemia; ALL, acute lymphoblastic leukemia; CR, complete remission; TNC, total nucleated cells; ATG, antithymocyte globulin; GVHD, graft-vs.-host disease; CSA, cyclosporine; MMF, mycophenolate mofetil*.

*^a^European Leukemia Net (ELN): ALL: t(9;22) or bcr/abl, t(4;11)/ALL-AF4, del(11q23), t(1;19), t(8;14), −7, +8, low hipoploidia (30–39 chromosomes), almost triploid (60–78 chromosomes), complex karyotype (≥3 chromosomal abnormalities). AML: monosomic karyotype, complex karyotype (≥3 chromosomal abnormalities), normal karyotype with FLT3-ITD^mut^, t(3;3), inv(3), t(9;22), t(6;9), -5/5q-, -7/7q-, 3q26 abnormalities, 11q23 abnormality [non t(9;11)]*.

*^b^On the basis of antigen-level HLA-A and HLA-B and allele level HLA-DRB1 typing*.

The institutional ethics committee for clinical research approved this study, and a written informed consent in accordance with the recommendation of the Declaration of Human Rights, the Conference of Helsinki, and institutional regulations was obtained from all patients.

### Study Groups

We have considered two groups of patients based on the incompatibility HLA-C1/2 between UCB and recipients:

– Group 1 (*n* = 21): the patient is homozygous, lacks any of the C ligands for inhibitory-KIR present in UCB NK cells and, therefore, is susceptible to develop NK-mediated GvL.
– Group 2 (*n* = 12): patients heterozygous C1/C2. As both inhibitory ligands are present, patients would not be susceptible to develop KIR-mediated GvL effect regardless the UCB compatibility.

### HLA Genotyping

Genomic DNA was extracted from peripheral blood samples drawn in EDTA anticoagulant tubes with Maxwell 16 Instrument to provide an automated method of purification of nucleic acids (Promega Corporation, Madison, WI, USA) and was typed for HLA class I alleles following the manufacturer’s instructions. HLA-C genotyping were performed with the INNO-LIPA HLA-C kits (Fujirebio Europe N.V., Gante, Belgium), using HLA specific primers for nucleic acid amplification of the different Loci. This is based on the PCR-SSO reverse method. Then, HLA alleles were determined with LIRASTM software for INNO-LIPA HLA.

SSP technique was used on samples that have ambiguity by SSO or for situations where higher resolutions were required. These analyses were made using HLA-C locus High Res SSP Unitray Kits (Invitrogen by life technologies corporation, Brown Deer, WI, USA).

In all patients, genotyping was done at a level of resolution, which allowed determining the KIR-binding epitope to distinguish the HLA-C dimorphism at position 80 of the α1 helix. In this case, HLA alleles were grouped into two major categories based on the amino acid sequence determining the KIR-binding epitope in HLA-C. HLA-C alleles are of the C1 or C2 group. HLA-C allotypes express C1 epitopes (characterized by an asparagine in position 80) or C2 epitopes (sharing a lysine in position 80). Although, two HLA-B alleles (HLA-B*46:01 and B*73:01) are considered as C1 group, none of our patients presented it (12).

### Transplantation Procedure

All patients underwent transplantation with a single unit UCB that was required to be HLA-matched with the recipient at ≥4–6 loci. Table 1 summarizes transplantation characteristics. Conditioning regimens, immune suppression, graft selection, and supportive care have been previously reported (13, 14). Briefly, the conditioning regimen was myeloablative in most of the patients (29 patients, 88%) comprising thiotepa 5 mg/kg for 2 days, busulfan 3.2 mg/kg for 3 days (dose was adjusted in patients weighing <34 kg), and fludarabine 50 mg/m^2^ for 3 days. Four patients (12%) received reduced-intensity conditioning consisting in fludarabine 30 mg/m^2^ for 5 days. Rabbit antithymocyte globulin (ATG) 2 mg/kg for 4 days was administered in all patients regardless the type of conditioning regimen. GVHD prophylaxis was based on cyclosporine combined with either long-course prednisone (76%) or mycophenolate mofetil (24%). The number of HLA disparities between UCB and recipients was not significantly different between the two groups. Cytomegalovirus status was reported in 31 patients (17 positive) and in 29 UCB donors (15 positive). Combined information is available in 29 patient–donor pairs, of which 6 were both negative and 8 were patient positive/donor negative.

### Transplant Outcome

We have studied the main variables of the transplant outcome that were the following: (1) probability of relapse (PR), defined as any morphologically proven recurrence of leukemia occurring after the allograft; (2) overall survival (OS), defined as the time from transplantation to death; and (3) event-free survival (EFS), time from transplantation to relapse or death from any cause, whichever comes first. Patients were censored at the time of relapse or of the last follow-up.

Secondary variables of analysis were engraftment, hematopoietic chimerism, non-relapse mortality (NRM), and acute or chronic GVHD. For the first one, we considered the incidence of neutrophil recovery as defined by a neutrophil count of a least 0.5 × 10^9^/L for three consecutive days and graft failure as no sign of neutrophil recovery as well as transient engraftment of donor cells within 60 days after transplantation. Full donor chimerism was defined as the presence of more than 95% of the donor cells, mixed chimerism if more than 5% and less than 95% were donor cells and autologous recovery if less than 5% of cells were donor cells. NRM was defined as deaths related to transplantation and not to relapse. Acute and chronic GVHD were diagnosed and graded according to the published criteria, with histopathological confirmation when possible, and evaluated in patients who survived at least 100 days with sustained engraftment (15, 16).

### Statistical Analysis

The Fisher exact test or chi-square test was used to compare categorical data and quantitative data were compared with the Mann–Whiney test. PR, OS, and EFS were calculated using the Kaplan–Meier method, including the 95% confidence interval (95% CI). The two-sided log-rank test was used for univariate comparisons. Factors associated with a *p-*value of 0.10 or less in univariate analysis and those with biologic significance were included in multivariate analysis. For multivariate analysis, we used Cox regression models, which were built using a stepwise forward/backward model procedure, and variables that attained *p* ≤ 0.05 were retained in the model. Cumulative incidence was used to estimate NRM, neutrophil and platelet recovery, and acute and chronic GVHD. Calculations were performed using IBM SPSS Statistical for Windows, version 17.0. (IBM Corp., Armonk, NY, USA).

## Results

The median follow-up time since the transplant for the surviving patients is 93 months (52–135 months). Table 2 shows the main outcomes according to incompatibility HLA-C1/C2 between UCB and recipients.

**Table 2:** Overall outcomes after unrelated cord blood transplantation.

Outcomes	Group 1 (%)	Group 2 (%)	*p*-Value univariate
Relapse at 5 years^a^	36 ± 13	84 ± 14	0.025
Overall survival at 5 years^a^	52 ± 11	17 ± 10	0.12
Event-free survival at 5 years^a^	43 ± 11	8 ± 8	0.09
Neutrophil recovery at day 25^b^	76 ± 9	67 ± 13	0.33
Platelet recovery at day 100^b^	83 ± 8	92 ± 8	0.72
Non-relapse mortality^b^	38	41	0.81
Acute GVHD II–IV incidence^b^	38	75	0.04
Chronic GHVD incidence^b^	14	36	0.16

*^a^Kaplan–Meier estimate*.

*^b^Cumulative incidence*.

*GVHD, graft-vs.-host disease*.

### Association of KIR-Ligand Absence in the GVH Direction with PR

**Figure 1:**
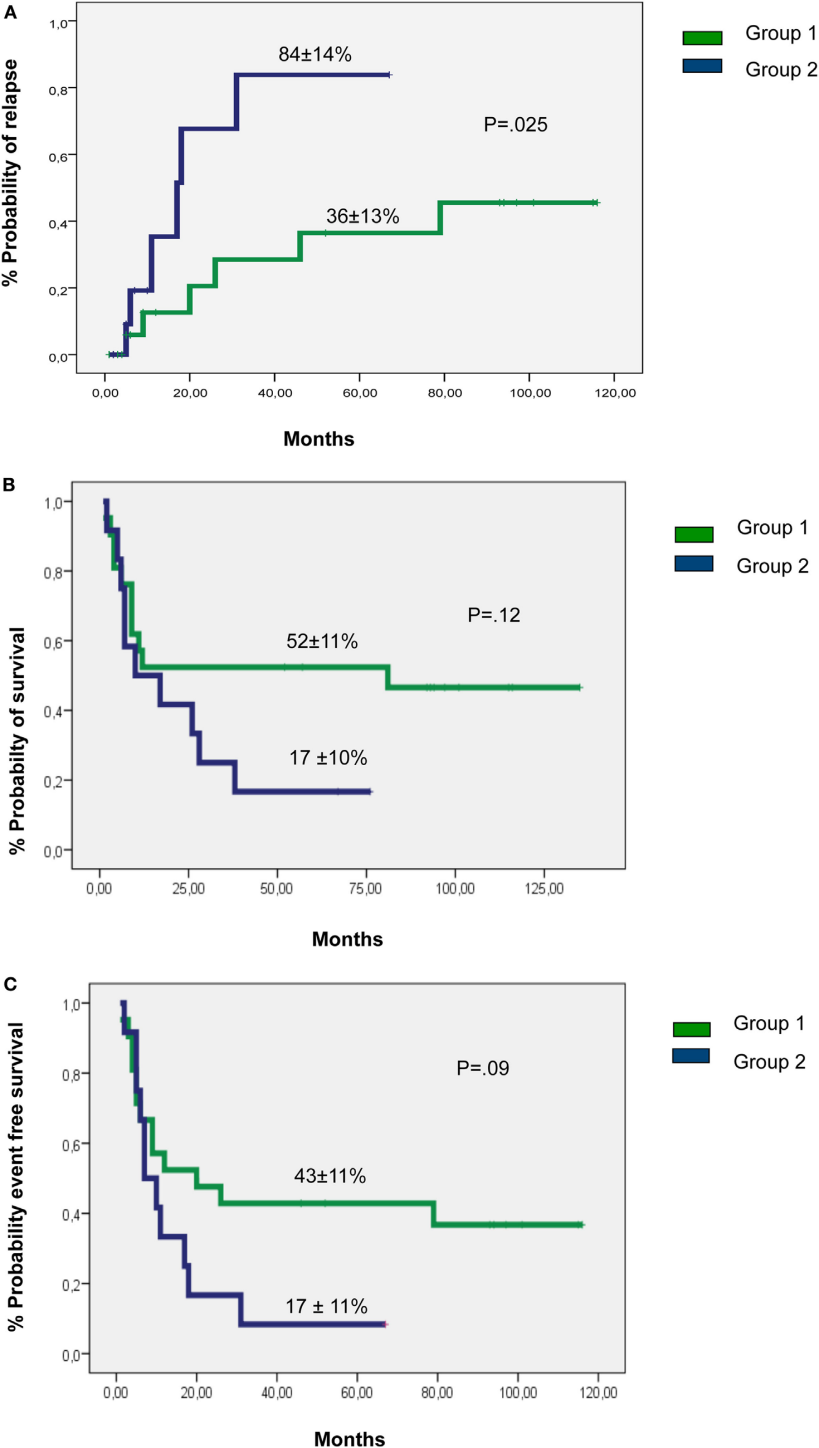
Probability of **(A)** relapse, **(B)** overall survival, and **(C)** event-free survival by groups 1 or 2.

The PR was increased in patients with absence of a C-ligand for inhibitory KIRs (group 1): the 2-year PR was 21 ± 10% for group 1 and 68 ± 18% for group 2 and the 5-year PR was 36 ± 13% for 1 and 84 ± 14% for 2 (*p* = 0.025) (Figure 1A).

This finding is also reproduced if we consider the subgroup of patients with diagnosis of ALL: the 2-year PR was 36 ± 21% for group 1 and 66 ± 26% for 2 (*p* = 0.038). In patients with diagnosis of AML, the 2-year PR was 41 ± 18% for group 1 and 50 ± 35% for 2 (*p* = 0.81).

In the univariate analysis, performed using demographic factors and transplantation variables such as sex, age (>14 years or ≤14 years), diagnosis (AML or ALL), status at transplant (first CR vs. >first CR), type of conditioning (myeloablative vs. reduced-intensity), KIR-ligand absence, and number of HLA disparities (6/6, 5/6 match vs. 4/6 match), the unique factor significantly associated with PR was KIR-ligand absence. In the multivariate analysis, the presence of KIR ligand was an independent risk factor associated with the increase in relapse probability [HR 3.750 (CI) 95% (1.095–12.838)] (*p* = 0.035), Table 3.

**Table 3:** Multivariate analysis.

	Relative risk	95% CI	*p*-Value
**Relapse**			
Sex	2.276	0.71–7.25	0.15
Age (≤14 vs. >14 years)	1.732	0.52–5.63	0.37
Acute myeloid leukemia (AML) vs. acute lymphoblastic leukemia (ALL)	1.81	0.44–7.33	0.41
Status at transplant (first CR vs. >first C)	0.746	0.33–4.75	0.7
HLA disparities (<2 HLA disparities vs. 2)	0.626	0.19–1.99	0.42
Conditioning regimen	0.531	0.06–4.12	0.51
Presence of KIR ligand vs. absence	3.750	1.09–12.8	0.03
**Overall survival**			
Sex	0.91	0.23–3.58	0.91
Age (≤14 vs. >14 years)	1.65	0.41–6.49	0.47
AML vs. ALL	0.31	0.05–1.75	0.1
Status at transplant (first CR vs. >first C)	1.00	0.24–4.08	0.99
HLA disparities (<2 HLA disparities vs. 2)	1.37	0.41–4.69	0.61
Conditioning regimen	2.01	0.41–10.1	0.39
Presence of KIR ligand vs. absence	5.77	0.79–41.6	0.08
**Event-free survival**			
Sex	1.94	0.85–4.34	0.11
Age (≤14 vs. >14 years)	1.51	0.66–3.42	0.33
AML vs. ALL	1.77	0.68–4.58	0.24
Status at transplant (first CR vs. >first C)	0.79	0.34–1.85	0.61
HLA disparities (≤2 HLA disparities vs. 2)	1.12	0.52–2.52	0.77
Conditioning regimen	0.58	0.13–2.51	0.58
Presence of KIR ligand vs. absence	1.99	0.87–4.57	0.11

### Association of KIR-Ligand Absence in the GVH Direction with OS and EFS

The 2-year OS was 52 ± 10% for group 1 and 50 ± 14% for 2, and the 5-year OS, 52 ± 11% for group 1 and 17 ± 10% for 2 (*p* = 0.12) (Figure 1B).

Event-free survival was better in patients of group 1: the 2-year EFS were 48 ± 11% for group 1 and 17 ± 11% for 2 and the 5-year EFS were 43 ± 11% for group 1 and 8 ± 8% for 2 (*p* = 0.09) (Figure 1C).

No factor was associated with OS and EFS in both univariate and multivariate analysis (Table 3).

### Association of KIR-Ligand Absence with Secondary Variables

#### Engraftment

The median time to myeloid engraftment was 19 days for the entire group (18 days for group 1 and 25 days for group 2). The probability of neutrophil recovery at day 25 posttransplant was 76 ± 9% for group 1 and 67 ± 13% for 2 (*p* = 0.33).

The median time to platelet engraftment was 42 days for the entire group (42 days for group 1 and 42.5 days for group 2). The probability of reaching platelet count greater than 20 × 10^9^/μL at day 100 was 83 ± 8% for group 1 and 92 ± 8% for 2 (*p* = 0.72).

Chimerism data were available in all patients. Complete donor chimerism was achieved in all patients at a median time of 43 days for the entire group. In group 1, the median time to achieve it was of 48 days, and in group 2, the median time was of 43 days (*p* = 0.51).

#### Acute and Chronic GVHD

At day 100, the cumulative incidence of grades II–IV acute GVHD for the entire population was 51% and it was significantly superior for group 2 (38% for group 1 and 75% for group 2, *p* = 0.04). The median time to the onset of grades II–IV acute GVHD was 45 days and the probability of development before day 45 was 44 ± 16% for group 1 and 56 ± 16% for 2 (*p* = 0.24).

The cumulative incidence of chronic GVHD for the entire population was 21% (14% for group 1 and 36% for group 2, *p* = 0.16). In group 1, the median time to develop chronic GVHD was 192 days and in group 2 was 169 days.

#### Non-Relapse Mortality

Non-relapse mortality for entire group was 39%. NRM was 38% for 1 and 41% for 2 (*p* = 0.81). The causes of death were infections (Group 1: 54% vs. Group 2: 40%), GVHD (Group 1: 9% vs. Group 2: 0%), relapse (Group 1: 27% vs. Group 2: 50%) and others (Group 1: 9% vs. Group 2: 10%) (*p* = 0.42).

## Discussion

This study demonstrates that in patients with acute leukemia transplanted with a single-unit UCB, the absence of a C-ligand of inhibitory KIR in patient cells is significantly associated with a decrease in the PR and reduces acute GVHD incidence. In multivariable analysis, the absence of C-ligand in the recipient was an independent risk factor associated to significant superior probability of remaining in complete remission [HR 3.750 CI 95% (1.095–12.838), *p* = 0.035]. These data did not change when only patients with ALL were analyzed, but this effect did not reach statistical significance when the AML patients were analyzed separately, probably due to the reduced number of patients. In contrast, there was no association with chimerism, neutrophil, platelet recovery, and NRM.

Our results suggest that a beneficial impact on the PR after UCBT mediated by NK alloreactivity between UCB vs. recipient could be observed not only associated with ligand mismatch but also when the patient lacks a C-ligand for the inhibitory KIR. Therefore, this type of transplant could be considered a good option for leukemia patients, including those diagnosed of ALL, without a family compatible donor who lacks a C-ligand, this is, patients homozygous C1 or C2.

The results presented here have the important value of the long follow-up of patients included. The fact that HLA-C was included later than other alleles in the routine HLA typing of the UCB units makes that the studies that include this ligand do not have such a long follow-up (17, 18). In contrast, we have been able to analyze the results obtained after a long period of time (93 months of median), which has allowed us to know that the absence of an inhibitory ligand not only improves the rate of relapse in the immediate posttransplant period but this effect is prolonged over time.

It is well known that UCB is a good choice as a source of hematopoietic progenitors, and it is a natural source of NK cells, as it has been demonstrated in different works. Nowadays, UCBT has become an alternative for patients lacking a compatible donor, as more incompatibilities are tolerated with this type of transplant (19–21). In this study, the maximum of incompatibilities between donor and recipient was two and no difference between the number of HLA mismatches and outcome of transplant was observed in the multivariate analysis.

In 2002, the Perugia group showed the importance of the NK alloreactivity between donor vs. recipient, which reduces the risk of relapse in patients with acute leukemia after haploidentical transplant (22). The demonstration of the efficacy of NK alloreactivity has been a significant progress in the field of transplantation, since it confirms the importance of innate immunity in addition to adaptive immunity (4–6, 22–25). However, other researchers provide contradictory data, probably because of the type of disease evaluated, the mode of transplantation, or the definition of KIR-alloreactivity used in each case (26–29). Although the correct way should be to study both KIR genes and their HLA-ligands, most groups only analyses the ligands, since it is faster, easier, and cheaper than doing so.

The KIR and HLA are inherited separately and the number of KIR genes present in each person is variable (30). Therefore, given an HLA ligand, we cannot always ensure the presence of its corresponding KIR and not all ligand incompatibilities would have to be associated to KIR-mediated NK alloreactivity effect. For example, in the case of KIR3DL1 (which binds to Bw4): even though this gene is present in a high proportion (90–95%) of people, it does not translate to protein in more than 2/3 of the subjects (31). This assumption has been avoided in our study since we have analyzed the ligands of those KIRs that are present in all people (KIR-2DL2/2DL3) or in the majority (2DL1 in 96%) (32–34).

We also find advantageous to focus on studying only C-ligands rather than other known ligands such as A3/A11, whose receptor is KIR3DL2. Although KIR3DL2 is present in 100% of the population, the binding between KIR3DL2 and A3/A11 is weak and requires EBV infection or reinfection, which is not frequent in most patients (35, 36). Consequently, the study of C-ligands would solve the problems due to misclassification of the groups, and would facilitate the sorting of patients: those in which KIR-mediated NK alloreactivity is expected and those in which not.

Other groups such as those in Minnesota or Boston did not find significant differences in the PR related to incompatibility of KIR or KIR ligands. However, the patients in these studies were not homogeneous and in addition, they used double unit UCBT and only part of them received ATG (37, 38). Our study concurs with the results of other authors because it underlines the importance of KIR-mediated NK alloreactivity in UCBT (8, 9, 25). It may be possible that the *in vivo* T-cell depletion of donor and patient secondary to ATG administration contributed to posttransplant expansion of functional NK cells and facilitated alloreactivity in the presence of a KIR-ligand incompatibility. In our series, the conditioning treatment included ATG to eliminate a large part of the T-cells. This fact as well as the presence of cord-naïve T-cells could have facilitated the NK alloreactivity observed in our study.

The main difference of our study is how patients were classified, by the absence of a C-ligand (group 1) or the presence of both C-ligands (group 2), instead of taking into account only the incompatibility of ligands. Recently, Sekine et al. (39) reported that only patients homozygous for HLA-C2 group alleles had a higher 1-year relapse rate and worse survival after UCBT than did HLA-C1/C1 or HLA-C1/C2 patients. Unlike our work, two UCB units were used in almost all the patients of this study and they use the dominant engrafting unit of the two to determine the KIR-ligand compatibility. There is no evidence that the algorithm for donor vs. recipient NK cell alloreactivity be the same in case of double cord. The analysis is to 1 year but, like in our work, the beneficial effect is not for incompatibility, but for the absence of the KIR ligand.

Our work may have a limitation. We have not considered neither the possible activating effect of the KIR 2DS1, whose ligand is also of the group C (C2), nor the previously discussed inhibitors 3DL1 and 3DL2. The activity of NK cells depends on many activator and inhibitor receptors and the final effect depends on the balance of all of them and not on a particular one. Notwithstanding, the inhibitory effects of the KIR ligands predominate over the activator ones as previously described, and the result is a net increase in the PR in group 2 (40–42).

In conclusion, single unit UCBT in patients with acute leukemia resulted in a lower incidence of relapse for those lacking a ligand of group C in comparison with those heterozygotes C1/C2. If these results are confirmed in a larger number of patients, the absence of a C-ligand could be considered as an important criterion for the selection of UCB units for hematopoietic transplantation.

## Ethics Statement

This study was carried out in accordance with the recommendations of research protocol from Junta de Andalucia with written informed consent from all subjects. All subjects gave written informed consent in accordance with the Declaration of Helsinki. The protocol was approved by the Comité de ética de investigación de Córdoba.

## Author Contributions

Conceived the study: CM-L, CM, RG, and BM. Collected the data: EG. Analyzed the data: CM-L and BM. Wrote the paper: CM-L and RG. Revised the manuscript critically: CM and CH. All authors approved the final manuscript.

## Conflict of Interest Statement

The authors declare that the research was conducted in the absence of any commercial or financial relationships that could be construed as a potential conflict of interest.
